# De-risking Pretreatment of Microalgae To Produce Fuels and Chemical Co-products

**DOI:** 10.1021/acs.energyfuels.4c00508

**Published:** 2024-04-30

**Authors:** Jacob S. Kruger, Skylar Schutter, Eric P. Knoshaug, Bonnie Panczak, Hannah Alt, Alicia Sowell, Stefanie Van Wychen, Matthew Fowler, Kyoko Hirayama, Anuj Thakkar, Sandeep Kumar, Tao Dong

**Affiliations:** † Renewable Resources and Enabling Sciences Center, National Renewable Energy Laboratory, 15013 Denver West Parkway, Golden, Colorado 80401, United States; ‡ BioSciences Center, National Renewable Energy Laboratory, 15013 Denver West Parkway, Golden, Colorado 80401, United States; § Catalytic Carbon Transformation & Scale-Up Center, National Renewable Energy Laboratory, 15013, Denver West Parkway, Golden, Colorado 80401, United States; ∥ Department of Civil and Environmental Engineering, Old Dominion University, Norfolk, Virginia 23529, United States

## Abstract

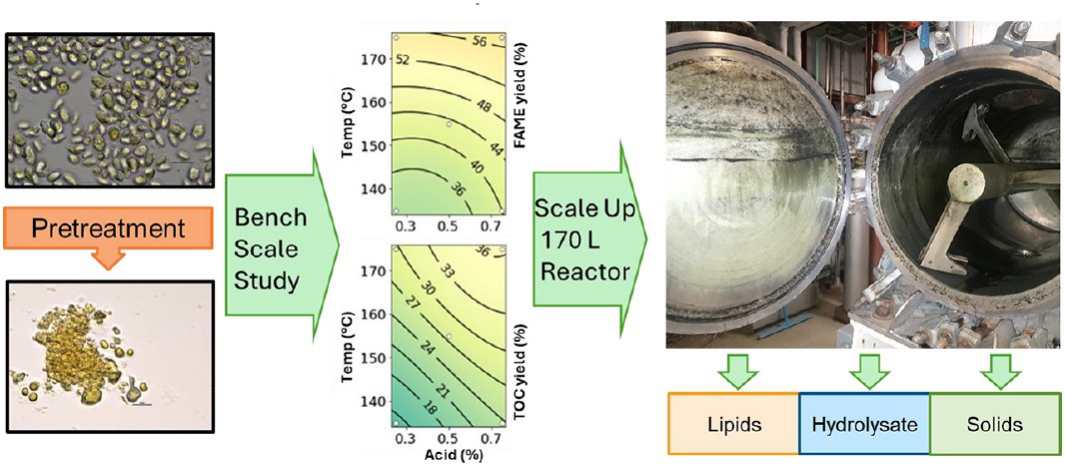

Conversion of microalgae to renewable fuels and chemical co-products by pretreating and fractionation holds promise as an algal biorefinery concept, but a better understanding of the pretreatment performance as a function of algae strain and composition is necessary to de-risk algae conversion operations. Similarly, there are few examples of algae pretreatment at scales larger than the bench scale. This work aims to de-risk algal biorefinery operations by evaluating the pretreatment performance across nine different microalgae samples and five different pretreatment methods at small (5 mL) scale and further de-risk the operation by scaling pretreatment for one species to the 80 L scale. The pretreatment performance was evaluated by solubilization of feedstock carbon and nitrogen [as total organic carbon (TOC) and total nitrogen (TN)] into the aqueous hydrolysate and extractability of lipids [as fatty acid methyl esters (FAMEs)] from the pretreated solids. A range of responses was noted among the algae samples across pretreatments, with the current dilute Brønsted acid pretreatment using H_2_SO_4_ being the most consistent and robust. This pretreatment produced TOC yields to the hydrolysate ranging from 27.7 to 51.1%, TN yields ranging from 12.3 to 76.2%, and FAME yields ranging from 57.9 to 89.9%. In contrast, the other explored pretreatments (other dilute acid pretreatments, dilute alkali pretreatment with NaOH, enzymatic pretreatment, and flash hydrolysis) produced lower or more variable yields across the three metrics. In light of the greater consistency across samples for dilute acid pretreatment, this method was scaled to 80 L to demonstrate scalability with microalgae feedstocks.

## Introduction

Algal biomass is a promising resource for producing renewable fuels and chemicals, but despite decades of research, algal biorefining for biofuel production remains in a pre-commercial state. While the cost of producing algal biomass is one primary hurdle to commercialization,^1−4^ the technology to convert the biomass to desired products is also in need of development. In particular, recent economic analyses have indicated that high-value co-products are necessary to offset the cost of fuel production if the fuel is to be sold at a price competitive with petroleum-derived fuels,^1−3^ and while many potentially suitable co-products have been identified,^5,6^ fewer have been demonstrated or validated.^7−11^ The slate of co-products available in a biorefinery also depends strongly upon the fuel production pathway of choice and upstream operations, especially pretreatment of algae biomass to make fuel and co-product precursors available for separation and conversion.^6^

One leading concept for algal biorefining is the parallel or combined algal processing (PAP or CAP) pathway, which fractionates algal biomass into an organic lipid phase, a fermentable aqueous hydrolysate, and a residual solid phase and then upgrades each phase using technology tailored to the chemistry of each fraction.^3,12,13^ A key aspect of the CAP approach is a pretreatment step, which lyses cells to render the lipids extractable while solubilizing carbohydrates and/or proteins into the fermentable hydrolysate. Historically, CAP has employed a dilute Brønsted acid pretreatment,^12−16^ which proved generally robust for high-carbohydrate and high-lipid algae. However, we hypothesized that alternative pretreatments, such as alkaline hydrolysis, flash hydrolysis,^7,17−19^ and enzymatic hydrolysis,^20−24^ may be more robust for high protein and/or variable composition biomass. Similarly, in developing alternative pretreatment approaches, we identified potential opportunities for process intensification. In particular, we hypothesized that pretreatment agents could assist in up- or downstream steps, flocculation of algae during harvest, or hydrolysis of lipids to allow for easier lipid fractionation and purification in polymer and fuel production.

To these ends, we identified six pretreatment approaches with the potential to compete with dilute Brønsted acid pretreatment across algae strains of highly variable composition, in terms of protein and carbohydrate solubilization while exposing the lipid fraction for easy extraction.

These approaches and their motivations are summarized in Table 1. Notably, this screening did not include pretreatments based on physical cell disruption (e.g., high-pressure homogenization, bead milling, and ultrasonication) because these techniques target mainly cell lysis, usually with minimal solubilization or deconstruction of carbohydrates and proteins, and, thus, are not as well-suited to a CAP approach favoring production of a fermentable hydrolysate with monomeric carbohydrates and amino acids during the pretreatment. Additionally, our previous experience with some of these techniques indicated severe emulsion formation that inhibited lipid extraction, even for high-lipid biomass, and we expected similar or increased emulsion formation with high-protein biomass.

**Table 1:** Summary of Pretreatment Approaches Applied to Microalgal Biomass in This Work

pretreatment	(potential) advantages	(potential) disadvantages
dilute Brønsted acid	baseline technology, proven on high-carbohydrate and high-lipid biomass	may not perform as well on high-protein biomass
agent: H_2_SO_4_ (up to 100 mg/g algae)		
temperature: 135–175 °C		
pressure: up to 150 psig		
time: 15 min		
dilute Lewis acid	FeCl_3_ may serve as both a flocculant for algae harvest and acid for pretreatment and may be recyclable	FeCl_3_ is more expensive than H_2_SO_4_
agent: FeCl_3_ (up to 100 mg/g algae)		
temperature: 135–175 °C		
pressure: up to 150 psig		recycling may increase process complexity
time: 15 min		
Twitchell Brønsted acid^25^	a lower temperature than baseline may allow for pretreatment without a pressure vessel	a longer time may require larger reactors that offset cost savings
agent: H_2_SO_4_ (100 mg/g algae)		
temperature: 80–120 °C	previously commercial technology for hydrolyzing fatty acid esters to free fatty acids may eliminate the need for NaOH-promoted saponification during lipid upgrading	acid loadings likely still necessitate more expensive metallurgy for industrial-scale reactors
pressure: up to 30 psig		
time: 8–16 h		
dilute alkali	NaOH may be used for both cell lysis and lipid saponification, eliminating one unit operation from biorefinery	NaOH has a higher cost and larger environmental footprint than H_2_SO_4_
agent: NaOH (up to 100 mg/g algae)	alkali might be more effective to hydrolyze biomass with a high protein content	some acid is still needed to protonate fatty acids prior to extraction
temperature: 135–175 °C		
pressure: up to 150 psig	alkali can also solubilize silica in diatoms that may be present in some feedstocks	
time: 15 min		
enzymatic hydrolysis	eliminates the need for expensive pretreatment pressure vessels	cost of enzyme cocktails may offset advantages
agent: enzymes (pH 5–7)		
concentration: up to 40 mg/g algae	enzyme cocktails targeted to algae components may allow for high levels of solubilization	long incubation times may require larger reactors that offset savings from mild conditions
temperature: 37–50 °C		
pressure: ambient	neutral range pH and low temperature and pressure	
time: 16–24 h		
flash hydrolysis	lyses cells without added chemical agents and in short residence time	hydrolysate may need additional conditioning to become fermentable
agent: none		
temperature, 180–240 °C		potentially higher CAPEX as a result of the higher operation pressure
pressure: 1000–1500 psig		
time: 10 s		low solid loading may limit industrial applications

## Materials and Methods

### Microalgal Strains

Nine samples of biomass were selected for pretreatment: *Scenedesmus acutus* LRB0401, *Scenedesmus* sp. IITRIND2, *Scenedesmus obliquus* UTEX393, *Monoraphidium minutum* 26BAM, *Picochlorum celeri* TG2, and *Tetraselmis striata* LANL1001 were cultivated in photobioreactors by Arizona State University (ASU) as part of the DISCOVR consortium,^35^ and these samples were received as a frozen slurry. *Nannochloropsis* sp. (dry biomass) was donated by an industry collaborator. Two mixed-culture samples (dry biomass) of wastewater-grown algal biomass were donated by separate industry collaborators, denoted as WWT1 and WWT2. WWT2 was provided by CLEARAS Water Recovery, Inc. (https://www.clearassolutions.com/) and typically consisted of three major genera of algae, *Chlorella*, *Scenedesmus*, and *Monoraphidium*, and picoplankton. The two wastewater-grown samples and *S. acutus* LRB0401 were grown in freshwater, and the other samples were grown in saltwater.

### Compositional Analysis

Composition of each algae sample was determined by National Renewable Energy Laboratory (NREL) laboratory analytical procedures (LAPs) for moisture, ash, carbohydrate, protein, and lipid [fatty acid methyl ester (FAME)] content.^26−28^ In addition, soluble and insoluble ash were distinguished by the following protocol. Biomass samples were ashed using the standard LAP for algae, and then duplicate ash samples were weighed out into 50 mL falcon tubes. Deionized water was added to each tube at a volume of 20 mL, and then samples were heated to near boiling in a water bath. Samples were then filtered through pre-combusted glass fiber filters, and an additional 40 mL of near boiling deionized (DI) water was poured over the sample and filtered. Filters were then dried at 40 °C under vacuum for 24 h before they were combusted at 575 °C, using the same ramping protocol in the algae LAP for moisture and ash. Soluble ash was then determined on the basis of the difference between the original ash weight and the weight of the ash sample after the above procedure was performed. The method is based on ISO 1576:1988 “Tea—Determination of Water-Soluble Ash and Water-Insoluble Ash”. Extracted solids were analyzed by the same protocols, except only the total ash was measured.

### Small-Scale Pretreatment Screening

To ensure a robust comparison, a range of conditions were selected, as described for each pretreatment. Wet biomass was diluted with DI water to make up a 15% (w/v) working stock slurry for pretreatment. Biomass working stocks were stored in freezers until needed. The screening experimental design was based on a central composite design (CCD) with a reduced number of experiments representing the four corner points of the experimental space, with a triplicate center point. This design was intended not as an optimization task but rather to establish a performance baseline across an expected reasonable range of operational conditions for each pretreatment. Pretreatment results were compared on the basis of lipid (as FAME) extractability and total organic carbon (TOC) and total nitrogen (TN) yields to the aqueous hydrolysate.

### Dilute Brønsted and Lewis Acid and Alkali Pretreatments

The three chemicals used were sulfuric acid (Brønsted acid), ferric chloride (Lewis acid), and sodium hydroxide (alkali). Biomass slurry (15%, w/w), DI water, and acid or base for 0.25, 0.5, or 0.75 wt % to produce a 5 mL total volume at 7.5% (w/w) algae solids, along with a rare earth metal stir bar, were sequentially loaded into a 10 mL CEM microwave tube. Each tube was heated to pretreatment temperature and held for 15 min (Table 2). After pretreatment, the biomass was allowed to cool to ambient temperature. Including heating and cooling, the total time above ambient temperature was approximately 22 min. To the alkaline-pretreated samples, a stoichiometric amount of acid was added to neutralize the alkaline samples plus 0.25 mL of acid for preservation before lipid extraction and analysis. TOC and TN analyses accounted for the extra dilution in these samples.

**Table 2:** Conditions for Brønsted and Lewis Acid and Alkali Pretreatment Screening

experiment	treatment (wt %) (H_2_SO_4_, FeCl_3_, or NaOH)	temperature (°C)
1	0.25	135
2	0.25	175
3	0.50	155
4	0.75	135
5	0.75	175

### Twitchell Pretreatment

Similar to the experimental design described above, five different conditions were applied to pretreat the algal biomass using the Twitchell approach (Table 3). To ACE glass 21 mL pressure tubes, 5 mL of 15% (w/w) biomass slurry, 0.75 mL of 10% sulfuric acid, 4.25 mL DI water and a small, rare earth metal stir bar were added. Tubes were placed in a heated oil bath for the duration of the pretreatment time. Triplicate ACE glass tubes were taped evenly together before being placed in the heated oil bath. After treatment, ACE glass tubes were then vortexed for 1 min and 5 mL of sample was aliquoted into a CEM microwave tube for workup and analysis.

**Table 3:** Conditions for the Twitchell Pretreatment

experiment	temperature (°C)	time (h)
1	80	8
2	80	16
3	100	12
4	120	8
5	120	16

### Enzymatic Hydrolysis

Similar to the pretreatments above, five different conditions were used to test enzymatic hydrolysis as a pretreatment process (Table 4). An enzyme mix was prepared to include lipase (Sigma L0777), phospholipase (Sigma L3296), Cellic Ctec3 (cellulase/hemicellulose, Novozymes), Chitinase (Sigma C6137), lysozyme (Sigma L6876), sulfatase (Sigma S9626), and DI water. A total of 2.5 mL of each biomass (15%, w/w, 375 mg) was aliquoted into seven 10 mL CEM microwave tubes. A total of 5 μL of the antibiotic nourseothricin (GoldBio N-500-100) was added to each tube to prevent microbial growth. Enzyme mix and DI water were aliquoted into each tube to provide 5, 13, and 40 mg/g algae biomass of each enzyme, hard-capped, and incubated at 30, 40, and 50 °C for 16 h at 225 rpm on shaker plates (Table 4). A protease mix was prepared of equal amounts of proteinase K (GreenBioResearch GPR10), trypsin (Sigma T1426), and papain (Sigma P3375). After incubation with the carbohydrate hydrolytic enzyme mix for 16 h, the protease mix and DI water were added to the sample tubes to equal 150 μL of additional volume and also equal 5, 13, or 40 mg/g algae biomass of each protease to match the previous enzyme loading rate. The tubes were then incubated at the indicated pretreatment temperatures for an additional 8 h before processing. Samples were checked periodically to verify that adequate mixing occurring in the shakers.

**Table 4:** Conditions for Enzymatic Hydrolysis Screening

experiment	temperature (°C)	enzyme mix (mL)	protease mix (μL)	mg of enzyme/g of algae biomass
1	30	0.333	20	5
2	50	0.333	20	5
3	40	0.833	50	13
4	30	2.5	150	40
5	50	2.5	150	40

### Flash Hydrolysis

Because of the larger volume required for flash hydrolysis, only four conditions were used to pretreat the algal biomass samples (Table 5). Experiments used either 240 or 280 °C at a backpressure of 1000 or 1500 psig, with a 7.5 wt % solid feed. The slurry was pumped into the reactor at 95 mL/min, producing a residence time of 10 s. Each run lasted 10 min, including the time required for stabilization of the pressure and temperature. Samples were collected for analysis only during a stable operation.

**Table 5:** Conditions for Flash Hydrolysis Pretreatment

experiment	temperature (°C)	pressure (psig)
1	240	1500
2	240	1000
3	180	1500
4	180	1000

### Sample Processing for TOC, TN, and Lipid Extraction

Each sample was vortexed and centrifuged at 750 relative centrifugal force (rcf) for 5 min to assist with phase separation. The top liquid was pipetted off, leaving the bottom residual solid phase, filtered through a 0.2 μm filter into a 15 mL centrifuge tube, preserved using 1 drop of 37% HCl, and stored in a 4 °C refrigerator for TOC and TN analyses.

To the remaining solid sample, 1 mL of 200 proof ethanol and 3 mL of hexane were added for lipid extraction.^29^ Sample tubes were placed on a 15-tube stir plate to stir overnight (∼15 h). The sample tubes were then vortexed and centrifuged at 750 rcf for 5 min, and the upper organic layer was carefully transferred by a Pasteur pipet into a pre-weighed 5 mL glass tube. Hexane was evaporated using nitrogen gas for approximately 20 min, before being placed in a 40 °C vacuum oven for 1 h. To the remaining biomass slurry, an additional 3 mL of hexane was added, and the sample tubes were vortexed and centrifuged at 750 rcf for 5 min. Once the 5 mL glass tubes containing the first organic extract were fully dried and weighed, the second organic layer extract in the remaining biomass slurry tubes was transferred to the associated 5 mL extraction glass tubes. Again, hexane was evaporated using nitrogen gas for 20 min, and the tubes were placed in the vacuum oven overnight. The tubes were weighed to obtain a total mass oil extraction yield. The oil was redissolved in 1 mL of methanol/chloroform solution (1:1), and 5–7 mg equiv of oil sample was transferred to a pre-weighed gas chromatography (GC) vial for FAME analysis.

### Contour Plot Generation

TOC, TN, and FAME extraction data from the small-scale screening experiments were fed into a machine learning algorithm to generate contour plots of the experimental space, similar that by Cao et al.^30^ The algorithm used a support vector model with a radial basis function with parameters γ = 0.15, ε = 0.5, and *C* = 20. For combinations of feedstock and pretreatment that we were unable to run as a result of operational issues (*Nannochloropsis* sp. and WWT1 samples at all conditions and *P. celeri* TG2 at the higher temperature condition in flash hydrolysis), yields were set to zero. For pretreatments where there was insufficient feedstock to run all data points (*M. minutum* 26BAM in flash hydrolysis), yields from the excluded data points were set to be equivalent to the one condition that was run. Finally, some enzymatic hydrolysis samples showed negative TOC and TN yields after subtracting the carbon and nitrogen contents added with the enzyme cocktail from the measured TOC and TN values. These negative values were assumed to be within experimental error of zero yield, and thus, yields were set to zero in these cases for the purpose of generating contour plots.

### Large-Scale Pretreatment

On the basis of the small-scale screening, the optimal pretreatment conditions selected were 2 wt % H_2_SO_4_, 175 °C, and 15 min reaction time. The larger scale run used a 160 L steam-injected Jaygo paddle reactor at 20 wt % solids. This run employed 21.6 kg of as-received *Nannochloropsis* sp. algae flake (73.52 wt % solids, 15.88 kg of dry cell weight equivalent), comprising 79.4 kg of total mass at 20 wt % solids. The algae flake was poured into the reactor from the top, and the feed chute was rinsed with 41.7 kg of DI water to produce a slurry slightly higher than 20 wt % solids. This slurry was stirred overnight at 150 rpm to rehydrate the algae flake. Then, 1.71 kg of H_2_SO_4_ was mixed with 4.76 kg of DI water and poured into the reactor, and the feed chute was rinsed with the remaining 9.6 kg of DI water to produce the desired concentrations of algae solids and H_2_SO_4_. The final H_2_SO_4_ concentration was 2 wt %, and the final algal solid concentration was 20 wt %. The reactor was heated indirectly with a steam jacket to 80 °C and then directly by steam injection to 175 °C. When it reached 175 °C, it was held for 15 min and then cooled by a combination of circulating chilled water and steam flashing. When the temperature decreased below 40 °C, the pretreated slurry was drained into 5 gallon polyethylene buckets and the reactor was rinsed sequentially with 25.95 and 12.75 kg of DI water.

### Large-Scale Solid–Liquid Separation

The pretreated, acidic slurry was stored at 4 °C overnight, during which time significant settling of the pretreated solids occurred, leaving a relatively non-turbid hydrolysate phase on top. The slurries were allowed to settle for another 3 days at 4 °C, although additional settling was minimal. After the solution settled, the hydrolysate phase was decanted into a fermentation vessel and additionally separated using an Alfa-Laval Clara 20 separator.

### Large-Scale Lipid Extraction

The separated solids were extracted for lipid recovery using a ratio of 3:1:3 solids/ethanol/hexane.^29^ Extractions were conducted in several batches using 1–2 kg of solids per batch and extracting each batch 3–6 times. Extractions were conducted at room temperature for 1 h per extraction, after which the mixture was centrifuged at 750 rcf, the top hexane layer was decanted, and the hexane layer was removed by rotary evaporation. The recovered hexane was recycled and used for subsequent extractions.

## Results and Discussion

### Compositional Analysis

The nine algae samples displayed a broad range of compositions (Table 6). The ash content ranged from 2.24% for *S. acutus* LRB0401 to 38.45% for the WWT1 sample. The protein content ranged from 11.33% for *S. acutus* LRB0401 to 47.51% for *S. obliquus* UTEX 393, corresponding to 2.37 and 9.94% total nitrogen, respectively. Similarly, the carbohydrate content ranged from 4.96 to 46.77%; the lipid content ranged from 2.05 to 24.64%; and the total carbon content ranged from 31.89 to 52.45%. The WWT1 and *S. acutus* LRB0401 samples provided the lower and upper bounds, respectively, for all three metrics.

**Table 6:** Compositional Analysis of the Nine Algae Samples

sample	pH	ash	protein	carbohydrate	lipid	total carbon	total nitrogen
*Nannochloropsis* sp.	6.72	25.62	32.22	6.88	8.49	40.89	6.74
WWT1	6.91	38.45	23.80	4.96	2.05	31.89	4.98
WWT2 (CLEARAS)	6.66	12.34	43.83	9.98	6.99	46.79	9.17
*T. striata* LANL1001	7.52	19.50	35.75	6.78	7.28	42.26	7.48
*S. acutus* LRB0401	5.40	2.24	11.33	46.77	24.64	52.45	2.37
*Scenedesmus* sp. IITRIND2	5.28	8.28	43.88	5.98	8.83	46.15	9.18
*S. obliquus* UTEX393	5.96	7.32	47.51	10.15	7.08	48.18	9.94
*M. minutum* 26BAM	5.37	6.65	41.06	11.06	9.30	49.87	8.59
*P. celeri* TG2	6.05	17.53	42.69	5.32	9.58	43.58	8.93

Almost all of the ash in each sample was insoluble ash (except for *P. celeri* TG2; Table 7). We expected that the saltwater-grown samples would have a higher ash content, but that does not appear to be the case. Saltwater-grown samples usually have a higher soluble proportion as a result of the salt in the growth media. It appears that the ash content in the harvested biomass is highly strain-dependent.

**Table 7:** Soluble and Insoluble Ash Contents of Algae Samples^a^

sample	total ash	insoluble ash	soluble ash	soluble (%)
***Nannochloropsis* sp.**	25.6	22.0	3.6	14.1
WWT1	38.5	35.1	3.4	8.8
WWT2 (CLEARAS)	12.3	11.1	1.2	10.0
***T. straita* LANL1001**	19.5	11.7	7.8	39.8
*S. acutus* LRB0401	2.2	2.0	0.3	12.5
***Scenedesmus* sp. IITRIND2**	8.3	5.6	2.7	32.4
***S. obliquus* UTEX393**	7.3	6.7	0.6	8.7
***M. minutum* 26BAM**	6.7	5.0	1.7	25.5
***P. celeri* TG2**	17.5	6.1	11.5	65.4

a Bold samples were cultivated in saltwater.

### Small-Scale Pretreatment Screening

**Figure 1:**
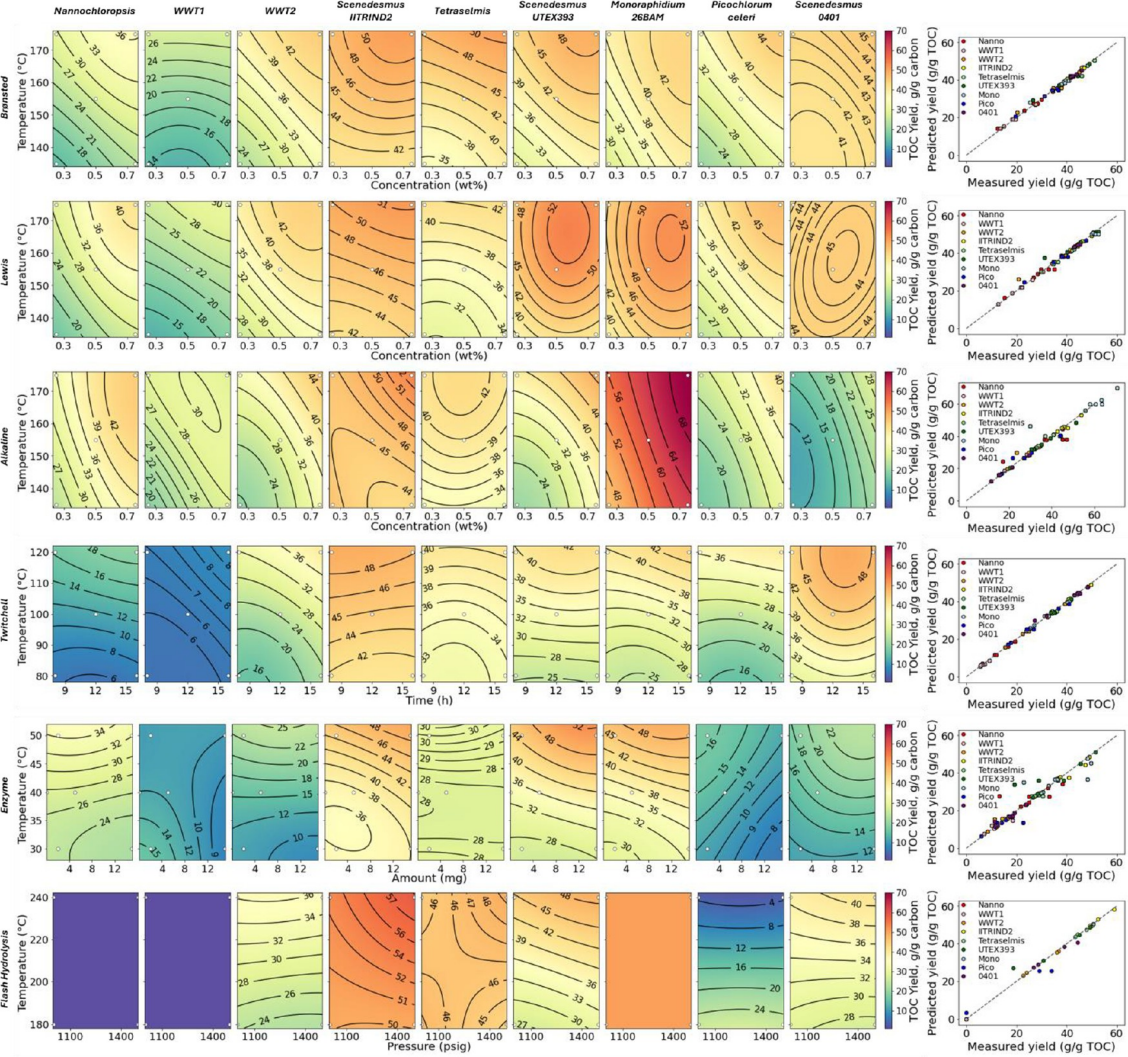
TOC yield to aqueous hydrolysate for each sample in a small-scale pretreatment screening. White dots indicate experimental points.

**Figure 2:**
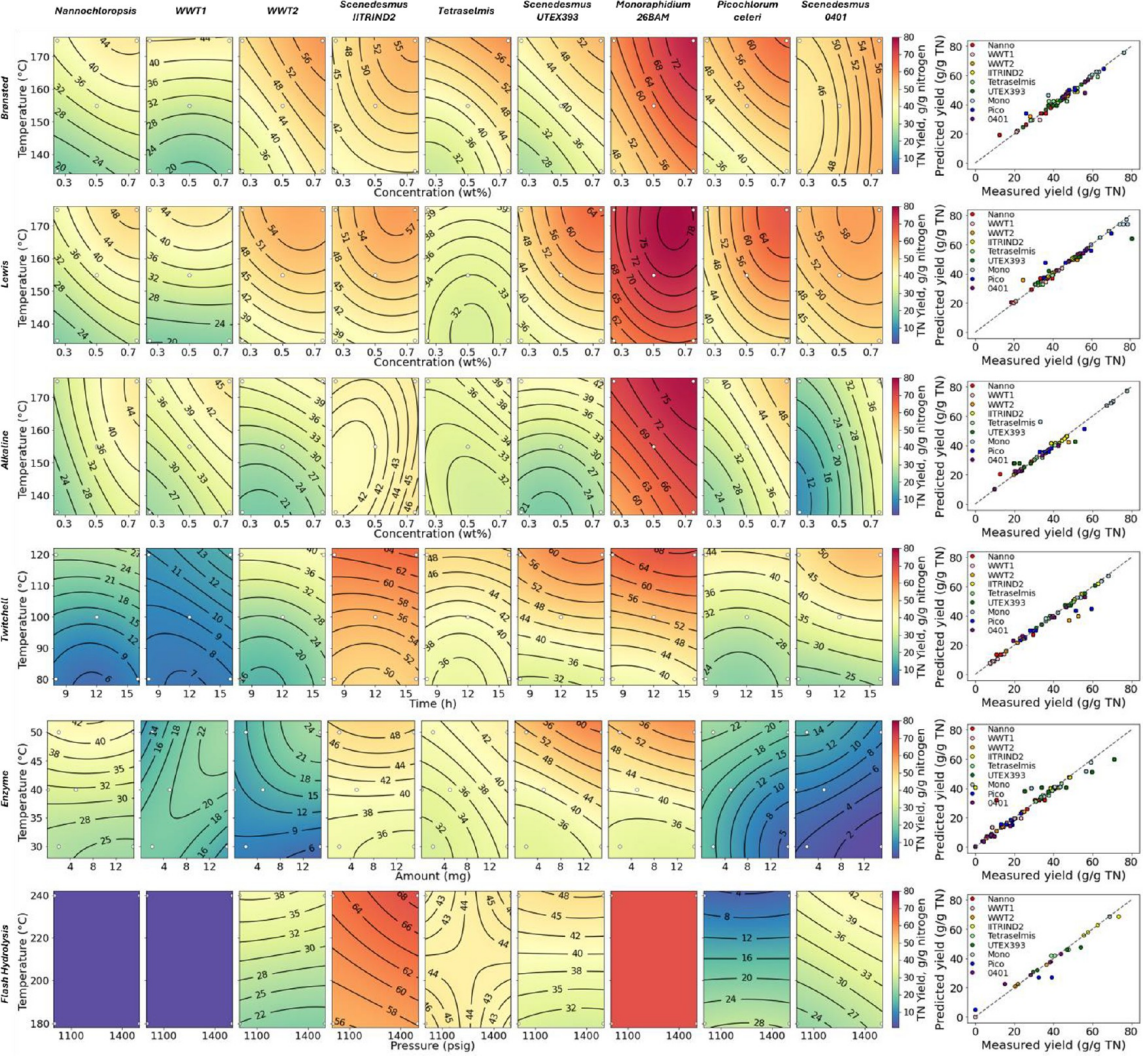
TN yield to aqueous hydrolysate for each sample in a small-scale pretreatment screening. White dots indicate experimental points.

**Figure 3:**
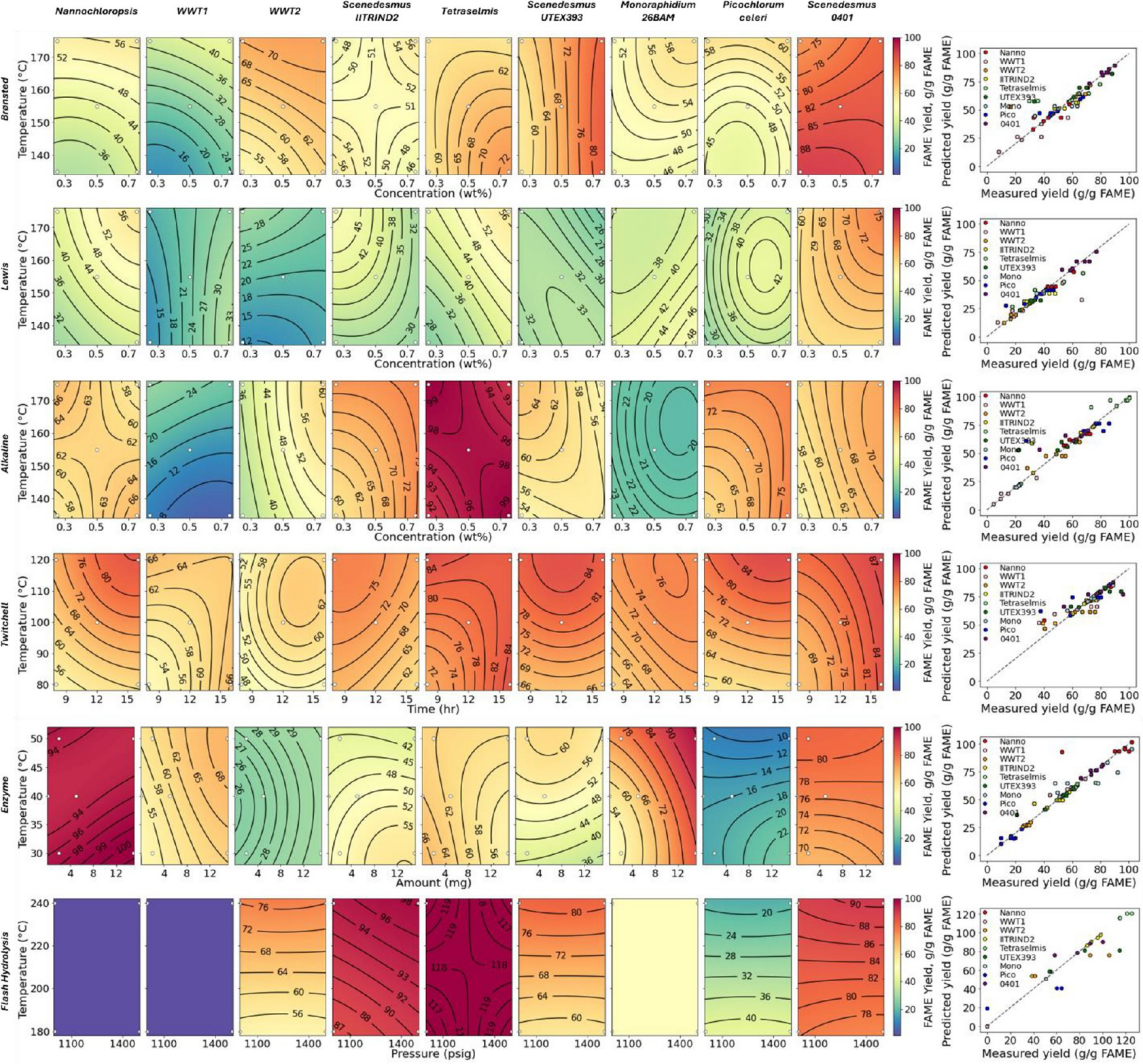
FAME extraction yield for each sample in small-scale pretreatment screening. White dots indicate experimental points.

Each algae sample was processed through five conditions (seven samples total) for five pretreatment approaches, representing 315 total data points. The samples were analyzed for the TOC and TN yields to the hydrolysate and lipid extraction yield as FAME from the residual solids. Figure 1 shows the compiled TOC yield results; Figure 2 shows the compiled TN yield results; and Figure 3 shows the compiled FAME yield results. In each of these figures, the contour plots serve mainly to facilitate a visual comparison across pretreatment approaches and algae species for a given metric and for the relative impacts of the two variables explored for each combination of pretreatment and algae species. That is, individual plots that are redder in color indicate an effective combination of algae species, pretreatment approach, and pretreatment conditions, while those that are more purple indicate an ineffective combination. Similarly, rows that contain generally redder plots indicate more effective pretreatment, and columns that contain generally redder plots indicate algae species that are more susceptible to a variety of pretreatments. The direction of the contour lines can give a qualitative idea of which of the two variables is more effective (e.g., more horizontal contour lines imply that the temperature is the more important variable, while diagonal contour lines imply that both variables are important), but the lines should not be overinterpreted. The parity plots in the far-right panels of each figure show that the models generally fit the experimental data well.

Carbon yields to the aqueous hydrolysate were mostly in the range of 35–50% for the dilute H_2_SO_4_ and FeCl_3_, although the *Nannochloropsis* sp. and WWT1 samples did not respond as well as the other samples to these treatments, probably as a result of the high ash buffering effect or encapsulation effect from drying. For a given algae sample, a higher temperature and acid loading tended to give higher carbon solubilization, although the effect was somewhat less pronounced for the FeCl_3_ samples. A similar trend held for the Twitchell dilute acid. The dilute NaOH treatment showed a broader range, with some samples producing yields similar to those of the dilute acid treatments, others producing lower yields, and some, in particular the *M. minutum* 26BAM sample, producing higher yields. In contrast, enzymatic hydrolysis generally produced a lower TOC yield than the other pretreatments. This was a surprising result given the variety and loading of enzymes added and underscores the need to better understand cell wall structures, which apparently contain motifs not easily accessed by common sugar- and protein-hydrolyzing enzymes. Flash hydrolysis produced hydrolysate TOC values that were generally competitive with or favorable to the other pretreatments at the most severe conditions but displayed some operational issues with some feedstocks. In particular, flash hydrolysis was unable to process the solid, initially dry samples because the biomass did not remain suspended in solution long enough to provide a consistent feed to the hydrolysis reactor. Flash hydrolysis also did not reach a steady state under the more severe conditions with *P. celeri* TG2 biomass. Finally, the available quantity of *M. minutum* 26BAM biomass only allowed for the collection of one data point at the most severe condition as a result of a limitation on the amount of biomass available.

Nitrogen yields to the hydrolysate followed trends similar to TOC solubilization and were generally slightly higher than carbon yields but showed more variability. The *Nannochloropsis* sp. and WWT1 samples that started from a dry state were generally lower than the other feedstocks. *M. minutum* 26BAM nitrogen was more prone to solubilization than the other feedstocks across most of the pretreatments. For the Twitchell pretreatment, similar to TOC solubilization, the temperature was more important than time. For enzymatic hydrolysis, both of the mixed-culture WWT samples along with the *P. celeri* TG2 and *S. acutus* LRB0401 samples showed low nitrogen solubilization, while the other two *Scenedesmus* samples showed relatively higher solubilization.

FAME yields were generally higher than TOC or TN yields, with enzymatic pretreatment of the WWT2 (CLEARAS) and *P. celeri* TG2 samples, NaOH treatment of the WWT1 and *M. minutum* 26BAM samples, and FeCl_3_ pretreatment in general standing out as exceptions. The low FAME yield for FeCl_3_ pretreatment, despite relatively high TOC and TN yields, may be a result of fatty acids forming insoluble Fe salts that are resistant to extraction and, thus, would be unavailable for extraction from the solids. It is also possible that the Lewis acid catalyzed the formation of fatty amides from fatty acids and free amine groups of proteins,^31^ because such amides may not have been detected in the FAME analysis. The low FAME yield for NaOH pretreatment of the WWT1 and *M. minutum* 26BAM samples, despite average or high TOC and TN yields, is counterintuitive. A potential reason may be that these samples were insufficiently acidified to protonate all of the free fatty acids (FFAs), because the FFA sodium salts partition more favorably to the aqueous phase. However, like the rest of the samples, these samples were acidified to pH 2 prior to extraction. Similarly, the FFA profile of these samples was not dramatically different from those of the other samples, suggesting that there must be some uncommon interaction of the other biomass components with NaOH in these samples. It is possible the fatty acids were converted to fatty amides under the alkaline conditions and, thus, were not detected as FAME, although it is not clear why this would be the case for only these two feedstocks. The low FAME yields for the WWT and *P. celeri* TG2 samples in enzymatic hydrolysis pretreatment are consistent with low TOC and TN yields, suggesting that the enzyme cocktail used here did not adequately match the cell wall chemistry of these samples and, thus, did not sufficiently disrupt the cell walls to release the lipids. Conversely, the Twitchell pretreatment produced relatively high FAME yields, despite lower TOC and TN yields, suggesting that cells were lysed but the long reaction times may have led to dehydration and/or condensation reactions that caused initially solubilized carbon and nitrogen to precipitate back out of solution. The *Nannochloropsis* sp. and WWT1 biomass similarly produced relatively high FAME yields in enzymatic hydrolysis, despite low TOC and TN yields, suggesting that drying the biomass may have made the lipids more extractable even without pretreatment, although the negative control enzymatic hydrolysis experiment lacking enzymes did produce lower FAME yields than those with enzymes.

**Figure 4:**
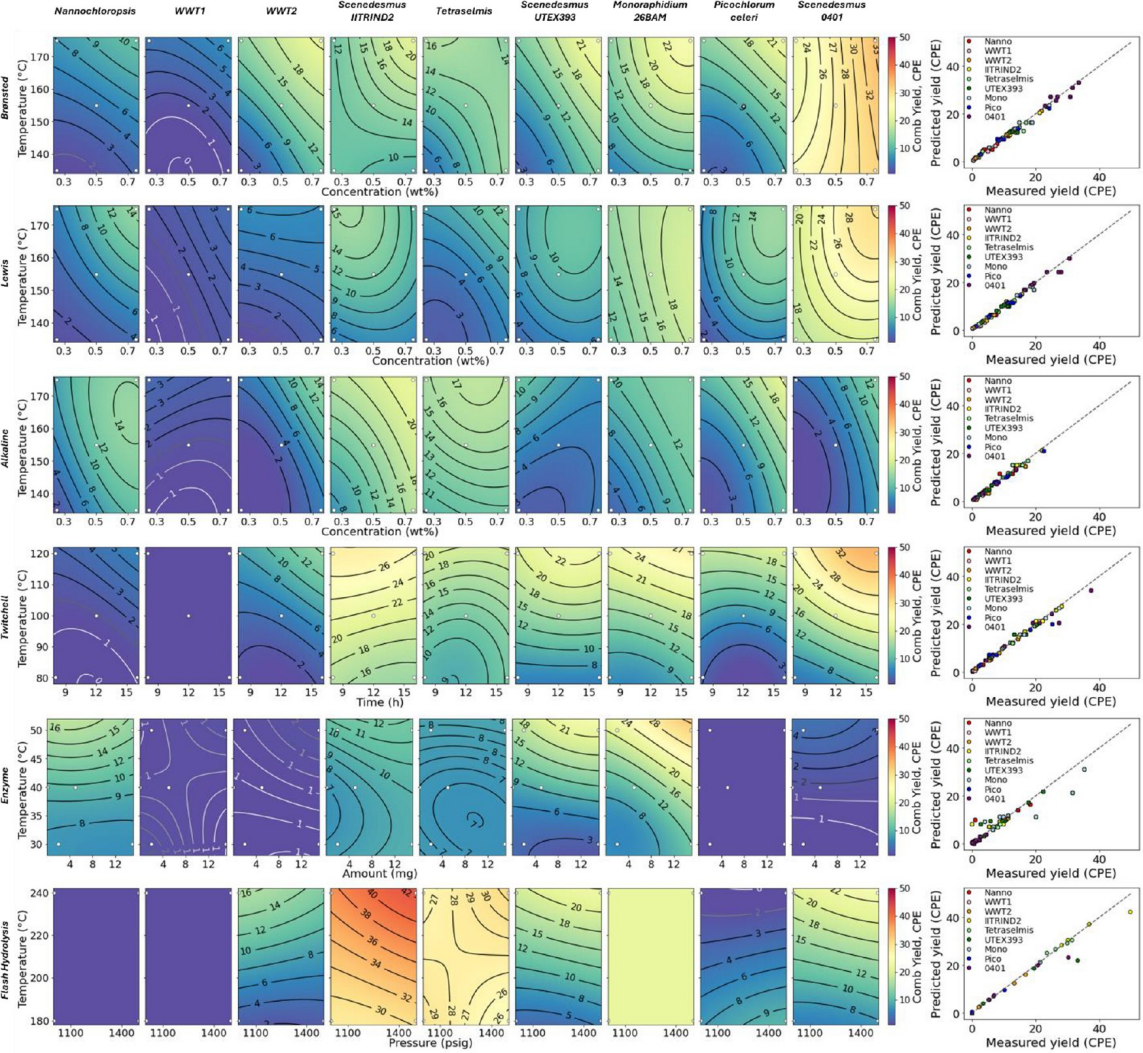
CPE for each combination of algae and pretreatment technology.

The intent of these experiments was to establish a baseline map of pretreatment effectiveness across algae strains and pretreatment technology rather than fully optimize each pretreatment or explain all of the observed trends at a molecular level. Thus, it is a useful exercise at this juncture to condense the screening data down to a single performance metric to compare the pretreatments in a more direct manner. In condensing the data, the weighting of the TOC, TN, and FAME metrics above will likely depend upon the downstream operations. In algae biorefining configurations utilizing fermentation of a hydrolysate, both carbohydrates and proteins are potentially desirable fermentation substrates,^12,32^ while lipids can contribute to both fuel and co-product streams.^8,33^ Thus, we have elected to weigh each metric equally and formulate a combined pretreatment effectiveness (CPE) for each combination of conditions and algae type by multiplying each factor, each of which ranges from 0 to 100%, together. Because FAME is expected to remain adsorbed to the residual solids until extraction, it does not contribute to the TOC yield, and a theoretical maximum TOC yield should instead be used. The theoretical TOC yield is calculated by subtracting the TOC represented in the FAME fraction from the total carbon in the biomass, as shown in eq 1. FAME does not contain nitrogen, and thus, the theoretical maximum is the same as the nitrogen content in the biomass. The CPE can then be calculated by eq 2.
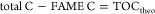
1
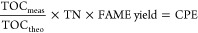
2As shown in Figure 4, *S. acutus* LRB0401 is amenable to acidic pretreatments under a variety of conditions. This is not surprising, because this sample is a nutrient-deplete, high-carbohydrate sample and acid pretreatment is well-known to be effective for algal polysaccharide hydrolysis.^13,14,29^ On the other hand, the WWT1 sample appears to be resistant to pretreatment under almost all conditions. The WWT1 sample had both high ash content, which may neutralize pretreatment agents, and heat treatment to meet standards for land application as a fertilizer. The heat treatment may have promoted cell agglomeration or condensation reactions between carbohydrates, proteins, and other components, leading to a lower accessibility of these components to the pretreatment agents. The remaining samples fall somewhere between these two extremes.

**Figure 5:**
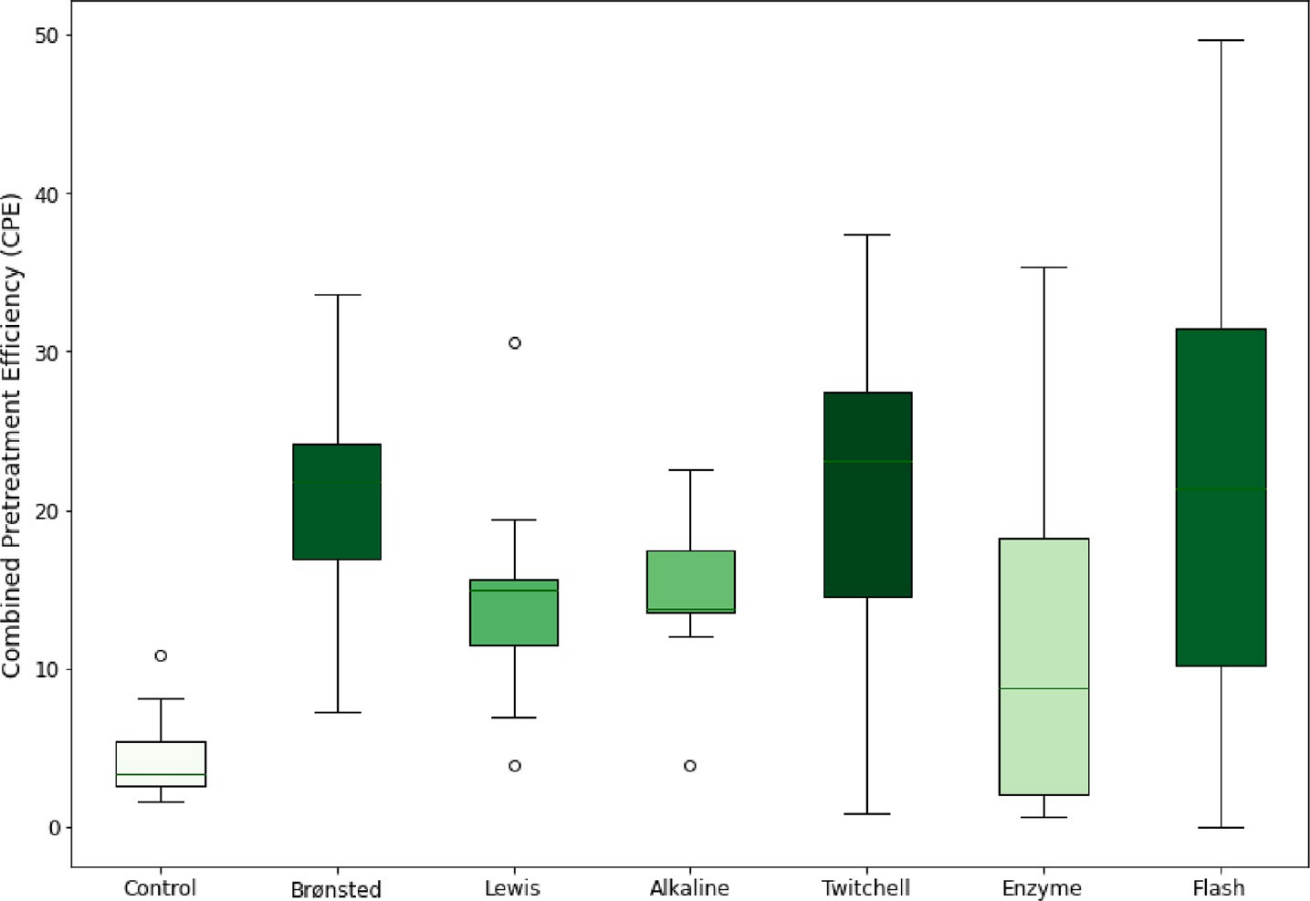
Best CPE across the algae samples.

To select a pretreatment to scale up, we compared the best CPE from each pretreatment and first evaluated which pretreatment had the highest average value across the algae samples. As shown in Figure 5, dilute H_2_SO_4_ (baseline or Twitchell conditions) and flash hydrolysis demonstrated the highest mean performance. The alkaline and FeCl_3_ pretreatments were slightly lower, while enzymatic hydrolysis was the lowest performing pretreatment. However, all pretreatments were better than the no pretreatment control. We also note that, for the Twitchell pretreatment, the best CPE was universally at the highest temperature (120 °C), indicating that it would still require a pressure vessel and, thus, negating one of the major potential benefits to this pretreatment approach over the baseline dilute acid pretreatment.

For algae grown in open ponds, seasonal temperature swings will necessitate crop rotation to the best species for a particular season.^34,35^ Thus, an ideal pretreatment should have an effective performance across multiple species to maintain a similar fractionation performance throughout the year. Among the top performing pretreatments by means, dilute Brønsted acid has the lowest variability. Of the technologies explored in this work, dilute Brønsted acid pretreatment gives the best combination of effective and consistent performance (Figure 5).

We do note, however, that the mean performance for most of the pretreatments is artificially lowered, while the range of performance is artificially increased by inclusion of the two solid samples (*Nannochloropsis* sp. and WWT1). However, excluding these two samples still leads to the same conclusion, namely, that dilute Brønsted acid pretreatment gives the best combination of effective and consistent performance.

### Large-Scale Pretreatment

With these results, we elected to perform dilute H_2_SO_4_ pretreatment at the 80 L scale and 20 wt % solids to evaluate the performance at more process-relevant conditions. For this run, we used the *Nannochloropsis* sp. sample and targeted the most severe conditions from the above screening, namely, 2 wt % H_2_SO_4_ (1:10 acid/biomass ratio), 175 °C, and 15 min, using a Jaygo reactor. The reactor took roughly 1 h to heat from room temperature to 175 °C, and the temperature was maintained at 172–174 °C for the duration of the pretreatment.

The reactor also took about 1 h to cool to 40 °C, at which point the contents were collected in buckets, including condensate from steam flashing and rinses. The condensate, rinsewater, and primary slurry were each kept separate. In total, 102 kg of primary slurry and 15 kg of condensate were collected, indicating that a little over 38 kg of water was added during the pretreatment as steam, of which about 23 kg remained in the slurry. After the reactor was drained of the primary slurry, the reactor was rinsed twice with water and reserved separately from the primary slurry for solid–liquid separation.

The slurry was initially foamy but settled into a relatively clear hydrolysate (∼60 vol %) and a solid phase (∼40 vol %) overnight in a refrigerator. Settling for 3 additional days did not significantly change the ratio. After the 4 days of settling, the hydrolysate phase was decanted and clarified with a continuous disc-stack centrifuge, producing 84 kg of clarified hydrolysate from the 102 L of primary slurry. The rinses were similarly clarified, recovering an additional 0.7 kg of solids from the clarifier, which were added to the primary solids. In total, 22.3 kg of wet solids were collected, at roughly 37 wt % (8.47 kg) dry solids content. Analysis of the primary hydrolysate showed a TOC content of 27.7 g/L and a TN content of 6.2 g/L, equivalent to a 43.5% TOC yield and 54.2% TN yield, respectively. These yields are slightly higher than those observed at the 5 mL scale, possibly as a result of the longer heating and cooling times at the 80 L scale.

### Lipid Extraction

An initial 50 g aliquot of solids was extracted to determine the extraction time needed to equilibrate the transfer of the lipid into the hexane phase. The extraction was mostly equilibrated after 1 h of mixing, and thus, a 1 h extraction time was used for the larger extractions.

Five aliquots of 1–2 kg of the separated solids were then extracted with ethanol/hexane (1:3, v/v), and the lipids were recovered from the hexane phase, as shown in Table 8. The first two aliquots were stirred magnetically, which did not mix the larger volumes of slurry and solvent as well as an overhead stirrer. Thus, for aliquots 3–5, overhead stirring was used, which resulted in higher lipid yields. In total, almost 6.5 kg of slurry was extracted, producing more than 500 g of extracted lipids and almost 6 kg of extracted solids.

**Table 8:** Lipid Extraction Summary

batch	input slurry (g)	solids (%)	total lipid extracted (g)
3	2030.4	37.3	187.6
4	973.6	37.3	99.3
5	1499.0	37.3	115.9
total	4503.0		402.8

The total algae feedstock was 15.88 kg of dry weight equivalent, at 8.49% FAME content, or 1.35 kg of total FAME. The pretreatment produced 22.3 kg of wet solids, which at 37.3% solids is equivalent to 8.31 kg of dry solids. These solids should contain all of the FAME. After two test batches to refine the extraction technique at the 5 L scale, we extracted an aliquot of 4.50 kg of wet solids (1.71 kg of dry solids), which theoretically contain 277.0 g of FAME total. Our crude lipid extract was 402.8 g total, and the FAME content of this extract was 44.7%, producing a FAME extraction yield of 65.0%. This FAME yield is slightly higher than the 57.9% yield achieved at the 5 mL scale, consistent with the TOC and TN measurements (Table 9).

**Table 9:** Comparison of the Pretreatment Performance at 5 mL and 80 L Scales

metric	5 mL scale	80 L scale
TOC yield	36.7	43.5
TN yield	45.5	54.2
FAME yield	57.9	65.0

The extracted lipids were also analyzed by solid-phase extraction for the content of neutral, polar, and free fatty acid fractions. By mass, the neutral lipid fraction, free fatty acid fraction, and polar lipid fraction accounted for 70.7, 20.5, and 8.7% of the lipid extract, respectively. However, FAME analysis revealed that the three fractions were 32.4, 57.3, and 14.7% FAME, respectively. Thus, the neural lipid fraction accounted for roughly 22% of the extracted FAME; the FFA fraction accounted for roughly 12%; and the polar fraction accounted for only 1% of the extracted FAME. Further analysis of the FAME (Table 10) showed that the FFA fraction was slightly enriched in saturated and monounsaturated lipids relative to polyunsaturated lipids, while the polar fraction was slightly enriched in unsaturated lipids relative to the neutral fraction.

**Table 10:** Analysis of Lipids Extracted from Solids Produced on the 80 L Pretreatment Scale

double bonds	neutral (%)	FFA (%)	polar (%)
0	33.4	38.2	28.1
1	47.0	51.2	49.3
2+	19.6	10.7	22.6
mass fraction	70.7	20.5	8.7
FAME purity	32.4	57.3	14.7

**Figure 6:**
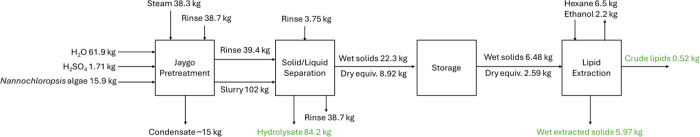
Mass balances for operations explored in this study.

The overall mass balance for the 80 L run is shown in Figure 6. There were small losses of solids in the condensate, in the clarifier, and in transferring between operations that are not shown, but the mass balance shows that 43.9% of the initial algae solids was solubilized into the aqueous phase, while 56.1% of the solids, including the lipid fraction, remained insoluble. Of those lipids, 65.0% could be extracted into a lipid phase at 44.7% FAME purity.

## Conclusion

We performed a small-scale screening of nine algae samples across five pretreatment approaches, reporting compositional analysis, TOC, TN, and lipid extraction data. These data showed that the baseline dilute H_2_SO_4_ pretreatment had an optimal balance of high pretreatment performance and low variability across the different algae strains, as measured by TOC, TN, and FAME yields. Operating at the 80 L scale, this pretreatment produced comparable TOC, TN, and FAME yields as at the 5 mL scale, indicating the scalability of this pretreatment approach. These results advance the field of algal biorefining by de-risking the pretreatment step and producing fractionated algae at a larger scale than has been previously demonstrated in the literature, thus allowing for subsequent de-risking of downstream operations.
